# Supplementary material for: Efflux-Related Carbapenem Resistance in Acinetobacter baumannii Is Associated with Two-Component Regulatory Efflux Systems’ Alteration and Insertion of ΔAbaR25-Type Island Fragment
Source: Int J Mol Sci. 2023 May 31;24(11):9525. doi: 10.3390/ijms24119525 (PMC10253819; doi:10.3390/ijms24119525)
Supplement: Supplementary file 1 [file ijms-24-09525-s001.zip › ijms-2421728-supplementary.pdf]

Supplementary Materials

"Efflux-Related Carbapenem Resistance in *Acinetobacter baumannii* is Associated with Two-component Regulatory Efflux Systems Alteration and Insertion of ΔAbaR25-type Island Fragment"

Alicja Słoczyńska, Matthew E. Wand, Lucy J. Bock, Stefan Tyski, and Agnieszka E. Laudy

Table S1. Epidemiological data, bla_{CHDL} gene groups and antimicrobial susceptibility profiles of clinical isolates of *A. baumannii* (n=61) to eight antimicrobial agents which are substrates for AdeABC and AbeM efflux pumps.

Groups of isolates carrying the following genes [9]		Isolate	Antimicrobial agent (interpretation of susceptibility results) [a]								Clinical material	Isolation year
			CAZ[b]	FEP	IMP	MEM	GEN	TOB	CIP	LEV		
$bla_{\text{OXA-51-like}}$	IS*Aba3*-$bla_{\text{OXA-58-like}}$	AB1	I	I	R	R	S	S	R	I	peritoneal fluid	2009
		AB3	I	S	R	R	S	S	R	I	urine	2009
		AB25	I	S	R	R	S	S	R	I	stoma swab	2010
		AB43	R	I	R	R	R	R	R	R	fistula	2010
		AB52	R	I	R	R	R	R	R	R	respiratory tract sample	2010
		AB62	I	I	R	R	S	S	R	I	wound swab	2011
	IS*Aba1*-$bla_{\text{OXA-23-like}}$	AB86	R	R	R	R	S	R	R	R	urine	2012
		AB87	R	I	R	R	R	R	R	R	urine	2012
		AB91	R	R	R	R	R	R	R	R	respiratory tract sample	2012
		AB92	R	R	R	R	S	S	R	R	respiratory tract sample	2012
		AB93	R	R	R	R	R	R	R	R	urine	2012
		AB96	R	R	R	R	R	R	R	R	respiratory tract sample	2012
		AB99	R	R	R	R	R	R	R	R	respiratory tract sample	2012
		AB111	R	R	R	R	S	R	R	R	urine	2012
		AB113	R	R	R	R	R	R	R	R	urine	2012
		AB118	R	I	R	R	S	S	R	R	rectal swab	2012
		AB119	R	R	R	R	S	R	R	R	stoma swab	2012
		AB129	R	R	R	R	S	S	R	R	urine	2014
		AB177	R	R	R	R	R	R	R	R	rectal swab	2013
		AB185	R	R	R	R	R	R	R	R	urine	2013
	$bla_{\text{OXA-24-like}}$	AB34	R	R	R	R	R	R	R	I	blood	2010
		AB37	R	I	R	R	R	R	R	R	wound swab	2010
		AB41	R	I	R	R	R	R	R	I	wound swab	2010
		AB42	R	R	R	R	R	R	R	R	respiratory tract sample	2010

	AB48	R	I	R	R	R	R	R	I	urine	2011
	AB76	R	I	R	R	R	R	R	I	wound swab	2011
	AB78	R	I	R	R	R	R	R	R	wound swab	2011
	AB79	R	I	R	R	R	R	R	R	fistula	2011
	AB81	R	I	R	R	R	R	R	I	wound swab	2012
	AB120	R	I	R	R	R	R	R	R	respiratory tract sample	2012
	AB121	R	R	R	R	R	R	R	I	blood	2012
	AB123	R	R	R	R	I	R	R	I	wound swab	2012
	AB124	R	I	R	R	R	R	R	I	respiratory tract sample	2012
	AB126	R	R	R	R	R	R	R	I	urine	2012
	AB127	R	I	R	R	R	R	R	R	wound swab	2012
	AB142	R	R	R	R	R	R	R	R	wound swab	2013
	AB145	R	S	R	R	I	I	R	R	fistula	2013
	AB149	R	I	R	R	R	S	R	R	wound swab	2013
	AB153	R	I	R	R	R	R	R	I	rectal swab	2013
	AB154	R	I	R	R	R	R	R	I	blood	2013
	AB156	R	I	R	R	S	I	R	I	urine	2013
	AB158	R	I	R	R	I	I	R	I	stoma swab	2013
	AB159	R	R	R	R	R	R	R	R	urine	2013
	AB161	R	I	R	R	R	R	R	I	respiratory tract sample	2013
	AB163	R	I	R	R	R	R	R	I	respiratory tract sample	2013
	AB165	R	I	R	R	R	R	R	I	respiratory tract sample	2013
	AB167	R	I	R	R	R	I	R	R	wound swab	2013
	AB168	R	I	R	R	R	S	R	I	respiratory tract sample	2013
	AB170	R	R	R	R	R	R	R	I	catheter tip	2013
	AB172	R	I	R	R	R	R	R	I	wound swab	2013
	AB174	R	R	R	R	R	R	R	I	respiratory tract sample	2013
	AB176	R	I	R	R	R	R	R	I	urine	2013
	AB180	R	R	R	R	R	R	R	R	urine	2013
	AB181	R	I	R	R	R	R	R	I	urine	2013
	AB183	R	I	R	R	S	I	R	R	wound swab	2013
	AB190	R	I	R	R	R	R	R	I	wound swab	2013
	AB192	R	I	R	R	R	R	R	R	surgical drain swab	2013
	AB193	R	I	R	R	R	R	R	R	respiratory tract sample	2013
	AB195	R	I	R	R	R	R	R	R	wound swab	2013
IS*Aba1*-*bla*$_{OXA-51-like}$	AB23	R	I	R	R	R	S	R	I	respiratory tract sample	2010
	AB140	R	S	I	R	R	R	R	R	rectal swab	2013

[a] Interpretation of MIC values of β-lactams as susceptibility of studied isolates were performed according to CLSI guidelines [54]. [b] CAZ, ceftazidime; FEP, cefepime; IMP,

imipenem; MEM, meropenem; GEN, gentamicin; TOB, tobramycin; CIP, ciprofloxacin; LEV, levofloxacin; R, resistant isolate; I, intermediate isolate; S, sensitive isolate.

Table S2. CarbAcineto NP test results and CCCP and PAβN effect on the carbapenem MIC values results for the bla_{CHDL}-carrying *A. baumannii* isolates (n=61).

Groups of isolates carrying the following genes [9]		Isolate	MIC (mg/L)						CarbAcineto NP test (time in minutes)
			IMP [a]	IMP +CCCP	IMP +PAβN	MEM	MEM +CCCP	MEM +PAβN	
$bla_{OXA-51-like}$	IS*Aba3*-$bla_{OXA-58-like}$	AB1	16	8	8	8	4	4	Uninterpretable [b] (120 min)
		AB3	8	4	4	8	4	4	Uninterpretable (120 min)
		AB25	8	4	4	8	4	4	Uninterpretable (120 min)
		AB43	16	1	8	64	32	16	Uninterpretable (120 min)
		AB52	16	8	16	8	4	4	Uninterpretable (120 min)
		AB62	16	8	8	8	4	4	Uninterpretable (120 min)
	IS*Aba1*-$bla_{OXA-23-like}$	AB86	16	8	16	16	16	16	Positive (45 min)
		AB87	16	8	4	32	16	8	Positive (45 min)
		AB91	16	8	8	16	16	8	Positive (45 min)
		AB92	16	8	8	32	16	8	Positive (15 min)
		AB93	16	8	16	32	16	16	Positive (45 min)
		AB96	32	16	16	32	16	8	Positive (25 min)
		AB99	16	16	16	16	8	8	Positive (100 min)
		AB111	32	16	16	64	32	16	Positive (60 min)
		AB113	32	16	8	64	32	8	Positive (45 min)
		AB118	32	16	16	64	32	16	Positive (60 min)
		AB119	16	8	16	64	32	16	Positive (60 min)
		AB129	8	8	4	8	4	4	Uninterpretable (120 min)
		AB177	16	8	16	64	32	16	Positive (75 min)
		AB185	16	8	8	8	8	4	Uninterpretable (120 min)
	$bla_{OXA-24-like}$	AB34	64	64	64	128	128	64	Positive (5 min)
		AB37	32	32	32	128	64	32	Positive (45 min)
		AB41	16	16	16	64	32	32	Positive (45 min)
		AB42	32	32	32	64	32	32	Positive (45 min)
		AB48	16	16	16	64	32	32	Uninterpretable (120 min)
		AB76	64	32	32	128	128	64	Positive (45 min)
		AB78	16	16	16	64	32	32	Positive (45 min)
		AB79	64	32	32	128	128	64	Positive (5 min)
		AB81	64	32	16	128	128	32	Positive (35 min)
		AB120	32	16	16	64	32	32	Uninterpretable (120 min)
		AB121	64	32	32	128	128	64	Positive (10 min)

	AB123	8	8	8	64	32	32	Uninterpretable (120 min)
	AB124	32	16	16	64	32	32	Uninterpretable (120 min)
	AB126	32	16	32	64	32	32	Positive (30 min)
	AB127	16	16	16	128	128	64	Positive (50 min)
	AB142	32	32	32	128	64	64	Positive (100 min)
	AB145	16	8	8	128	64	64	Positive (100 min)
	AB149	16	8	8	64	32	32	Positive (100 min)
	AB153	32	8	16	64	32	32	Positive (100 min)
	AB154	16	8	8	64	32	32	Positive (100 min)
	AB156	16	16	16	128	64	64	Positive (50 min)
	AB158	32	16	32	128	64	64	Positive (50 min)
	AB159	32	32	32	32	32	32	Positive (55 min)
	AB161	64	64	32	128	64	64	Positive (5 min)
	AB163	64	32	32	128	64	64	Positive (10 min)
	AB165	32	16	16	128	64	32	Positive (25 min)
	AB167	64	32	32	128	128	64	Positive (5 min)
	AB168	16	16	16	128	64	64	Positive (20 min)
	AB170	16	16	16	64	32	32	Positive (45 min)
	AB172	16	16	8	32	16	16	Positive (45 min)
	AB174	64	64	32	128	64	64	Positive (5 min)
	AB176	64	64	64	128	128	128	Positive (5 min)
	AB180	16	16	16	64	32	32	Positive (45 min)
	AB181	64	32	64	128	64	64	Positive (5 min)
	AB183	8	8	8	64	32	32	Positive (100 min)
	AB190	16	16	16	64	32	32	Positive (100 min)
	AB192	16	8	16	64	32	32	Uninterpretable (120 min)
	AB193	32	8	16	64	32	32	Positive (100 min)
	AB195	32	16	16	128	128	16	Positive (100 min)
IS*Aba1*-*bla*$_{OXA-51-like}$	AB23	8	8	8	8	8	8	Uninterpretable (120 min)
	AB140	4	4	4	16	16	8	Uninterpretable (120 min)

[a] IMP, imipenem; MEM, meropenem; CCCP, an efflux pump inhibitor Carbonyl cyanide 3-chlorophenylhydrazone; PAβN, an efflux pump inhibitor Ph-Arg-β-naphthylamide. [b] Uninterpretable result - compared to the internal control (red color tube), after the required incubation time (maximum 2 h), only the development of a red-orange color was observed in the test tube, but not the expected yellow or orange colors.

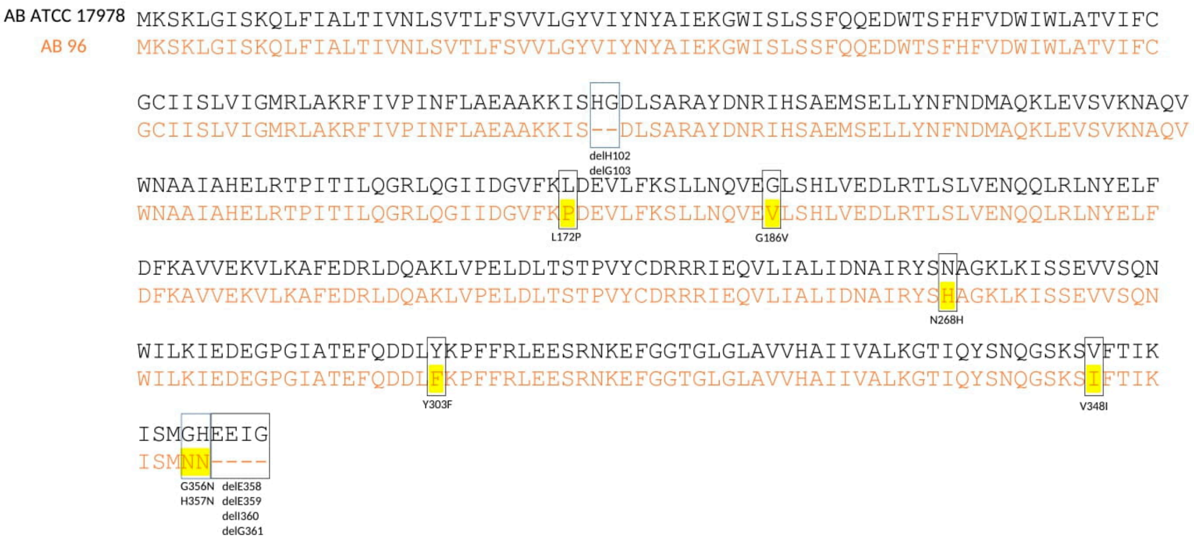

Figure S1. Sequence comparison of the AdeS protein of clinical isolate *A. baumannii* 96 (in orange font) and reference strain *A. baumannii* ATCC 17978 (in black font). The amino acid changes in AdeS are shown in the black boxes and additionally highlighted on a yellow background. The positions of the amino acid changes are given according to the AdeS protein of the reference strain.

Methods

Genome assembly

Raw nanopore data was basecalled using Guppy v6.1.3 in super accuracy mode (Oxford Nanopore Technologies, Oxford, UK). After quality filtering using NanoFilt [62] and residual adapter removal using Porechop (https://github.com/rrwick/Porechop) obtained dataset was quality checked using NanoPlot [62]. Long read assembly was performed using Trycycler pipeline [63]. In brief nanopore reads were initially assembled using four long read assemblers: flye, unicycler, Raven and miniasm. Long reads assemblies were next reconciled, circularized and final consensus was generated followed by polishing with racon and medaka. Long read assembled contigs were further polished using polypolish [64,65] and POLCA [66].

The remaining ambiguities in the genome assembly were verified by the PCR amplification of DNA fragments, followed by Sanger sequencing with an ABI3730xl Genetic Analyzer (Life Technologies) using BigDye Terminator Mix v. 3.1 chemistry (Life Technologies). All of the possible sequence errors and missassemblies were further manually corrected using Seqman software (DNAStar) to obtain complete nucleotide sequence of bacterial genome.

References

(The numbering of references cited in supplementary materials is consistent with the reference list in the main article.)

9. Słoczyńska, A.; Wand, M.E.; Tyski, S.; Laudy, A.E. Analysis of bla_{CHDL} genes and insertion sequences related to carbapenem resistance in *Acinetobacter baumannii* clinical strains isolated in Warsaw, Poland. *Int. J. Mol. Sci.* **2021**, *22*, doi:10.3390/ijms22052486.
54. Clinical and Laboratory Standards Institute M100: *Performance standards for antimicrobial susceptibility testing. 32nd ed.* CLSI; Wayne, PA, USA: **2022**
62. De Coster, W.; D'Hert, S.; Schultz, D.T.; Cruts, M.; Van Broeckhoven, C. NanoPack: visualizing and processing long-read sequencing data. *Bioinformatics* **2018**, *34*, 2666-2669, doi:10.1093/bioinformatics/bty149.
63. Wick, R.R.; Judd, L.M.; Cerdeira, L.T.; Hawkey, J.; Meric, G.; Vezina, B.; Wyres, K.L.; Holt, K.E. Trycycler: consensus long-read assemblies for bacterial genomes. *Genome Biol.* **2021**, *22*, 266, doi:10.1186/s13059-021-02483-z.
64. https://github.com/rrwick/Polypolish - accessed on 10.05.2023
65. Wick, R.R.; Holt, K.E. Polypolish: Short-read polishing of long-read bacterial genome assemblies. *PLoS Comput. Biol.* **2022**, *18*, e1009802, doi:10.1371/journal.pcbi.1009802.
66. Zimin, A.V.; Puiu, D.; Luo, M.C.; Zhu, T.; Koren, S.; Marcais, G.; Yorke, J.A.; Dvorak, J.; Salzberg, S.L. Hybrid assembly of the large and highly repetitive genome of Aegilops tauschii, a progenitor of bread wheat, with the MaSuRCA mega-reads algorithm. *Genome Res.* **2017**, *27*, 787-792, doi:10.1101/gr.213405.116.
